# Impact of faecal DM excretion on faecal calcium losses in dogs eating complete moist and dry pet foods – food digestibility is a major determinant of calcium requirements

**DOI:** 10.1017/jns.2017.11

**Published:** 2017-04-24

**Authors:** Ellen Kienzle, Thomas Brenten, Britta Dobenecker

**Affiliations:** 1 Department of Veterinary Sciences, Chair of Animal Nutrition and Dietetics, LMU München, Oberschleißheim, Germany; 2 Mars Petcare Europe, Verden, Germany

**Keywords:** Calcium requirements, DM digestibility, Faecal calcium losses, Dogs, BW, body weight, NRC, National Research Council

## Abstract

The recommendations for the Ca supply for maintenance of dogs have been reduced by about 75 % in the last decades. An important factor for Ca requirements is faecal Ca losses. In previous studies with experimental diets faecal Ca losses depended on Ca intake and on faecal DM excretion. A predictive equation for faecal Ca losses in mg/kg body weight (BW) developed in a fibre model is: faecal losses = −33·8 + (13·6 faecal DM excretion (g/kg BW)) + (0·78 Ca intake (mg/kg BW)). The present study aimed at testing this equation in pet food with material from trials carried out for other purposes. Digestion trials with twenty-five dry and fifteen moist foods (326 observations in total) were evaluated retrospectively. Faecal DM excretion and faecal Ca losses were significantly correlated (*r*^2^ 0·86; *P* < 0·001). There was a highly significant correlation (*r*^2^ 0·87; *P* < 0·001) between the experimentally determined faecal Ca excretion and the faecal Ca excretion predicted by the equation of Kienzle *et al*. The data from the previous fibre model study could be transferred to prepared moist and dry dog food. Faecal DM excretion has a considerable impact on faecal Ca losses in a practical feeding situation. In conclusion, Ca requirements for maintenance may vary with food DM intake and digestibility.

Recommendations for Ca supply of adult dogs for maintenance have undergone enormous changes over the last decades. In 1974 the National Research Council (NRC) recommended a Ca supply of approximately 3·6 g for a 15 kg dog^(^^1^^)^. The German Society of Nutritional Physiology (GfE) recommended less than half of this amount^(^^2^^)^, and in 2006 the NRC reduced the recommended intake of a 15 kg dog to approximately 1 g Ca^(^^3^^)^, and the minimum requirements to half of this. The NRC discusses many uncertainties in the bioavailability of Ca in dogs^(^^3^^)^. A recent meta-analysis^(^^4^^)^ plotted literature data on Ca intake against faecal Ca excretion in adult dogs in a modified Lukas test. The relationship between intake and faecal excretion showed a remarkable uniformity, a result which does not suggest a pronounced high variability of Ca availability. In trials with a low Ca intake of ≤30 mg/kg metabolic body weight (BW) there was, however, a high variation of faecal Ca excretion which ranged from 2 to 75 mg/kg metabolic BW. The data with very low faecal Ca excretion came exclusively from diets with meat and/or fat and/or cooked starch. These diets are likely to have a very high digestibility^(^^3^^)^. This suggests that digestibility of DM may affect faecal Ca losses. In a previous study with a fibre model Kienzle *et al.*^(^^5^^)^ showed that in diets with the same Ca intake faecal Ca excretion increased with faecal DM excretion, i.e. with a decrease in DM digestibility due to fibre addition. A predictive equation was established with the independent variables faecal DM excretion and Ca intake and the dependent variable faecal Ca losses on a BW basis. The predictive equation for faecal Ca losses in mg/kg BW was: faecal losses = −33·8 + (13·6 faecal DM excretion (g/kg BW)) + (0·78 Ca intake (mg/kg BW)) (equation 1). In the present study we investigated whether this equation from a fibre model^(^^5^^)^ would be suitable to predict faecal Ca losses in pet food from Ca intake and faecal DM excretion in a retrospective evaluation of an already existing dataset from a pet food digestion trial. The hypothesis was, that if this was true, then faecal Ca losses and consequently Ca requirements of dogs would vary with DM digestibility and food DM intake.

## Materials and methods

Standard procedure digestion trials (3 d dietary change to test diet, 7 d adaptation, 5 d trial, dogs separated for feeding, one meal per d, food intake measured daily, food allowance aimed to maintain BW according to historical data, dogs individually housed during 5 d trial for total faecal collection, water *ad libitum*, ≥2 weeks break between trials) with twenty-five dry and fifteen moist complete and commercially produced pet foods (Table 1) were carried out. A total of forty dogs (10–35 kg BW, nineteen beagles and twenty-one foxhound-crossbreds, age 1·5–5 years, thirty-three females, seven males, majority of animals intact, all products tested in both sexes, males in more trials than females, only one breed per trial) took part in the study. In total there were 326 single observations on Ca intake and faecal excretion (275 in beagles and forty-seven in foxhound-crossbreds). All procedures were approved by the Regierung von Oberbayern, the appropriate authority in Bavaria. For each trial the dogs were adapted to the food for a total of 10 d. This was followed by total faecal collection for 5 d. Faeces were lyophylised and ground. Ca was determined by flame photometry after wet digestion. Ca intake, faecal Ca and faecal DM excretion were calculated per kg BW to allow the application of the predictive equation of Kienzle *et al*.^(^^5^^)^ (equation 1). Linear regression analysis was carried out for Ca intake and faecal Ca excretion as well as between the experimentally determined faecal Ca excretion and the faecal Ca excretion as predicted by equation 1. A multiple linear regression with the independent variables faecal DM excretion and Ca intake and the dependent variable faecal Ca losses was calculated with the pooled data from the fibre model^(^^5^^)^ and the present study. All regressions were calculated using Sigmaplot 12.5 (Systat Software GmbH).

**Table 1.** Crude nutrient content of diets (% DM)

	Protein	Fat	Fibre	Ash	N-free extract
Mean	28·7	19·5	2·6	8·7	40·5
Range	20·0–46·4	8·9–41·5	0·5–7·5	6·1–15·0	11·5–60·0

## Results

**Fig. 1.**
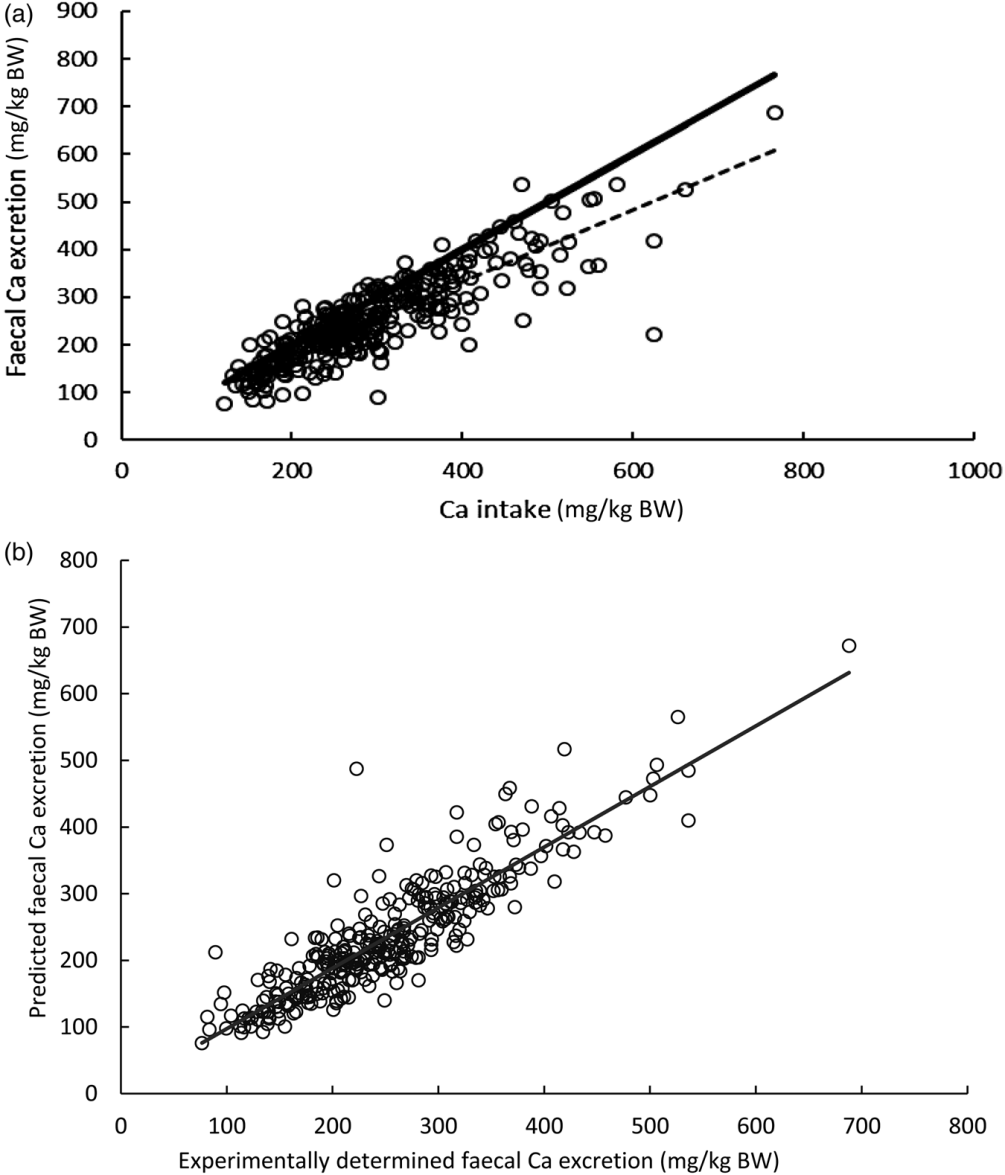
(a) Relationship between calcium intake and faecal calcium excretion. ----, Regression line: *y* = 31·66 + 0·75*x*; sem 45·7; *r*^2^ 0·741, *n* 326; ––––, *x* = *y*. (b) Relationship between experimentally determined and predicted faecal Ca excretion (by use of equation 1). ––––, Regression line: *y* = 6·47 + 0·91*x*; sem = 33·6; *r*^2^ 0·80; *n* 326. BW, body weight.

Ca intake and faecal Ca losses correlated significantly (*r*^2^ 0·86; *P* < 0·001; Fig. 1(a)). Even though Ca intake was clearly above the current recommended intake in all experiments there was a considerable number of trials with negative apparent Ca digestibility (see dots above the *X* = *Y* line in Fig. 1(a)). There was a highly significant correlation (*r*^2^ 0·87; *P* < 0·001) between the experimentally determined faecal Ca excretion and the faecal Ca excretion predicted by the equation of Kienzle *et al*.^(^^5^^)^ (equation 1) as shown in Fig. 1(b). The regression equation calculated from the pooled data of the present study and the investigation of Kienzle *et al*.^(^^5^^)^ between the independent variables faecal DM excretion (g/kg BW) and Ca intake (mg/kg BW) and the dependent variable faecal Ca losses (mg/kg BW) was: faecal Ca excretion = −9·4 + (0·7 Ca intake) + (16·8 DM excretion) (equation 2).

## Discussion and conclusions

The results of the present study clearly show that the results of the previous study in a fibre model^(^^5^^)^ can be transferred to prepared pet food. Faecal DM excretion has a strong impact on faecal Ca excretion also in commercially produced pet food. The findings of the present study may help to explain discrepancies between studies on Ca metabolism in dogs due to the choice of diet. Renal and cutaneous Ca losses are very low in dogs^(^^2^^)^. If these losses are not considered, a zero balance of Ca (i.e. maintenance of body Ca) would be achieved if faecal losses equal intake. Assuming this is the case, equation 2 can be rearranged to calculate the amount of Ca intake required for zero Ca balance in relation to faecal DM excretion and model calculations can be carried out for varying DM intake and DM digestibility. For example, if a dog with 15 kg BW eats 20 g DM/kg BW and the DM digestibility of the food is 75 %, a zero balance of Ca would be maintained at a Ca intake of 3·7 g. This agrees excellently with the recommendation of the 1974 NRC^(^^1^^)^. On the other hand, if the DM digestibility is 90 % and DM intake 20 g/kg BW, the amount of Ca which would then maintain body Ca in a 15 kg dog is only 1·2 g, which is rather close to the recommended allowance of the 2006 NRC^(^^3^^)^. These model calculations clearly show that DM digestibility and DM intake have to be considered for the recommendations for Ca allowance.

## References

[1] National Research Council (1974) Nutrient Requirements of Dogs. Washington, DC: National Academies Press.

[2] Ausschuß für Bedarfsnormen der Gesellschaft für Ernährungsphysiologie (1989) Energie- und Nährstoffbedarf: Nr. 5 Hunde *(*Energy and Nutrient Requirements: no 5 Dogs*)*. Frankfurt: DLG Verlag.

[3] National Research Council (2006) Nutrient Requirements of Dogs and Cats. Washington, DC: National Academies Press.

[4] Mack JK, Alexander LG, Morris PJ (2015) Demonstration of uniformity of calcium absorption in adult dogs and cats. J Anim Physiol Anim Nutr 99, 801–809. 10.1111/jpn.12294 25808498

[5] Kienzle E, Dobenecker B, Wichert B (2006) Effect of fecal water and dry matter excretion on fecal mineral excretion in dogs studied in a fiber model. J Nutr 136, 2001S–2003S. 16772479 10.1093/jn/136.7.2001S

